# A phase II trial of lomeguatrib and temozolomide in metastatic colorectal cancer

**DOI:** 10.1038/sj.bjc.6604366

**Published:** 2008-05-13

**Authors:** O A Khan, M Ranson, M Michael, I Olver, N C Levitt, P Mortimer, A J Watson, G P Margison, R Midgley, M R Middleton

**Affiliations:** 1 CR UK Medical Oncology Unit, Churchill Hospital, Old Road, Oxford OX3 7LJ, UK; 2 Department of Medical Oncology, University of Manchester, Christie Hospital, Wilmslow Road, Manchester M20 9BX, UK; 3 Department of Hematology and Medical Oncology, Peter MacCallum Cancer Centre, Melbourne, Victoria, Australia; 4 Royal Adelaide Hospital, Adelaide, South Australia, Australia; 5 Kudos Pharmaceuticals Ltd, 327 Cambridge Science Park, Milton Road, Cambridge CB4 0WG, UK; 6 CR UK Carcinogenesis Group, Paterson Institute for Cancer Research, Wilmslow Road, Manchester M20 4BX, UK

**Keywords:** *O*^6^-methylguanine-DNA methyltransferase, lomeguatrib, temozolomide, colorectal cancer, DNA repair

## Abstract

To evaluate the tumour response to lomeguatrib and temozolomide (TMZ) administered for 5 consecutive days every 4 weeks in patients with metastatic colorectal carcinoma. Patients with stage IV metastatic colorectal carcinoma received lomeguatrib (40 mg) and TMZ (50–200 mg m^−2^) orally for 5 consecutive days every 4 weeks. Response was determined every two cycles. Pharmacokinetics of lomeguatrib and TMZ as well as their pharmacodynamic effects in peripheral blood mononuclear cells (PBMC) were determined. Nineteen patients received 49 cycles of treatments. Despite consistent depletion of *O*^6^-methylguanine-DNA methyltransferase in PBMC, none of the patients responded to treatment. Three patients had stable disease, one for the duration of the study, and no fall in carcinoembryonic antigen was observed in any patient. Median time to progression was 50 days. The commonest adverse effects were gastrointestinal and haematological and these were comparable to those of TMZ when given alone. This combination of lomeguatrib and TMZ is not efficacious in metastatic colorectal cancer. If further studies are to be performed, emerging data suggest that higher daily doses of lomeguatrib and a dosing period beyond that of TMZ should be evaluated.

Colorectal cancer is the second most common cause of death in cancer patients in the Western world (Lieberman, 2006). Current treatment for colorectal cancer is dependent on the stage of the disease and is usually in the form of surgery, radiotherapy or chemotherapy. The most commonly used chemotherapy is a combination of 5-fluorouracil (5-FU) and leucovorin (1990). In stage IV metastatic disease, this enables survival for just over 1 year (Thirion *et al*, 2004). More recently, studies have been conducted in this patient group using oxaliplatin and irinotecan combined with 5-FU and leucovorin (de Gramont *et al*, 2000; Douillard *et al*, 2000). Sequential use of these agents has improved median survival to greater than 20 months, but response rates for second- or third-line treatments in metastatic colorectal cancer remain low (Tournigand *et al*, 2004; Saletti and Cavalli, 2006).

Temozolomide (TMZ) is an oral alkylating agent with broad spectrum of antitumour activity and relatively low toxicity (Payne *et al*, 2005). It has been shown to inhibit the growth of human tumour xenografts *in vitro* and is active in human colorectal lines (Friedman *et al*, 1995; Liu *et al*, 1996). Cytoxicity of TMZ is thought to be mediated principally through the methylation of DNA at the *O*^*6*^ position of guanine (Tisdale, 1987; Baer *et al*, 1993; D'Atri *et al*, 1995). Efficacy against colorectal cancer has not been extensively studied in clinical trials. In a phase I study of TMZ, 12 out of 22 patients had metastatic colorectal cancer and there was one partial response in this group, suggesting considerable tumour resistance to treatment (Spiro *et al*, 2001).

Tumour cell resistance to *O*^6^-alkylating agents is conferred by the DNA repair protein *O*^6^-methlylguanine-DNA alkyltransferase (MGMT) (D'Incalci *et al*, 1988; Pegg, 1990). This removes the alkyl group from guanine in a stoichiometric autoinactivating reaction before subsequent rounds of replication can give rise to apoptosis. Cell death after TMZ treatment also depends upon recognition of *O*^6^-guanine/thymine mispairs by the mismatch repair (MMR) pathway (Souliotis *et al*, 1991). Tumour cells frequently express a high level of MGMT. Treatment with *O*^*6*^-alkylating drugs is likely to be more effective when they are used in combination with drugs that inactivate the repair protein.

Lomeguatrib is a nontoxic low-molecular weight pseudosubstrate that has the ability to inactivate MGMT (Dolan *et al*, 1986, 1990). When used in combination with TMZ, lomeguatrib has been shown to sensitise human tumour xenografts of the methylating agent (Middleton *et al*, 2002). A phase I dose escalation study previously conducted using lomeguatrib/TMZ combinations demonstrated that intravenous administration of 10 mg m^−2^ lomeguatrib decreased levels of AGT in peripheral blood mononuclear cells (PBMC) and all tumours by over 95% (Ranson *et al*, 2006). Consistent and complete depletion of MGMT activity required 40 mg lomeguatrib when the drug was administered orally – after 20 mg only 56% of patients showed total depletion in PBMC.

Lomeguatrib was well-tolerated up to 80 mg m^−2^ in the phase I study (twice the proposed dose level in the current study), with no clear toxicity associated with the administration of the drug alone (Ranson *et al*, 2006). As consistent and complete depletion of MGMT activity was achieved with 40 mg oral lomeguatrib, a higher dose was not considered necessary in this trial. Consequently, we did not consider that dose reductions in lomeguatrib were required. Toxicity observed with the lomeguatrib/TMZ combination was qualitatively similar to that observed with TMZ alone. One patient with colorectal cancer experienced a >50% drop in carcinoembryonic antigen (CEA) in the course of treatment in the phase I trial. With 40 mg day^−1^ lomeguatrib p.o., the maximum-tolerated dose of TMZ was 125 mg m^−2^, and these were the starting doses for the current study.

Preclinical studies and the phase I trial provide evidence to suggest that the combination of lomeguatrib and TMZ could improve upon the modest activity of the methylating agent alone. The primary study objective was to evaluate tumour response rates to lomeguatrib and TMZ in patients with metastatic colorectal cancer, with secondary aims of describing time to progression and the safety and tolerability of the combination. Other objectives were to evaluate the biochemical response using CEA levels and to describe the pharmacokinetic and pharmacodynamic effects of lomeguatrib.

## MATERIALS AND METHODS

### Patient selection

Patients with histologically proven metastatic colorectal cancer were eligible for the study, provided that they had not previously received more than two systemic chemotherapy regimens. Other requirements included measurable disease; age >18 years; Eastern Cooperative Oncology Group performance status of 0 or 1; life expectancy >12 weeks; adequate bone marrow and biochemical function (haemoglobin >10 g dl^−1^, white blood cells >3 × 10^9^/l, absolute neutrophil count >1.5 × 10^9^/l, platelets >100 × 10^9^/l); creatinine ⩽1.25 upper limit of normal (ULN); bilirubin ⩽1.25 ULN; AST ⩽5 (metastases to liver) or 2 × ULN.

Patients were excluded if within 4 weeks of previous therapy; pregnant or nursing; still recovering from surgery; considered poor medical risks due to a serious, uncontrolled medical disorder, nonmalignant systemic disease or active, uncontrolled infection; had known CNS metastases, had a history of seizures, were on antiepileptic medication or had previously received an *O*^6^-alkylating agent.

The study was conducted in accordance with the principles of the International Conference on Harmonisation of Good Clinical Practice guidelines and the Declaration of Helsinki. The trial was approved by an independent ethics committee according to national and local requirements at each trial centre. All patients gave informed written consent.

### Study design and statistical considerations

This was a multi-centre open study to determine the response rate to lomeguatrib and TMZ. We aimed to recruit 30 patients with inoperable stage IV metastatic colorectal cancer. The sample size was selected on the basis that 30 patients would ensure that the standard error of the observed response rate was less than or equal to 0.1 and permit a satisfactory estimate of response rate, but incorporated an early stopping rule according to the method of Gehan, with the response rate of interest set at 20%. Descriptive statistics were generated for efficacy, toxicity, pharmacokinetic and pharmacodynamic end points. The median time to progression was estimated using Kaplan–Meier survival curves. Patients who had not progressed by the end of the study or who withdrew prior to progression were censored for the analysis.

### Drug administration

Lomeguatrib enteric-coated 10 mg capsules were obtained from Kudos Pharmaceuticals (Cambridge, UK), and TMZ purchased from Schering Plough Ltd (Welwyn Garden City, UK) as 5, 20, 100 and 250 mg capsules.

Patients received lomeguatrib 40 mg day^−1^ p.o. for 5 consecutive days every 4 weeks for up to six cycles. Temozolomide was administered at 125 mg m^−2^ day^−1^ p.o. 2 h after lomeguatrib. Patients fasted for 1 and 2 h before and after TMZ and lomeguatrib respectively.

Retreatment was permitted if the absolute neutrophil count was ⩾1.5 × 10^9^/l, the platelet count ⩾75 × 10^9^/l and any other toxicity had resolved to grade I or better. A treatment delay of up to 2 weeks was allowed for resolution of drug-related toxicity. Dose reductions in TMZ were mandated in the event of grade IV haematological toxicity, grade III toxicity lasting 7 or more days or any grade III or IV nonhaematological toxicity. These were in increments of 25 or 50 mg m^−2^ day^−1^ according to the type of toxicity encountered. The need for doses of TMZ below 75 mg m^−2^ day^−1^ required the patient to be removed from the study. Patients could be also withdrawn from the study for progressive disease, serious violation of the study drug protocol or withdrawal of consent.

### Evaluation of response and toxicity

All eligible patients who received any part of the treatment were considered assessable for response and toxicity. Patients were assessed for adverse events at each attendance. Physical exam, performance status and vital signs were recorded at the beginning of each treatment cycle. Complete blood count was checked prior to treatment and on days 14, 21 and 28, with blood chemistry tested on days 1, 14 and 28. Adverse events were graded according to the National Cancer Institute-Common Toxicity Criteria (NCI-CTC) version 2.0. Tumour response was assessed every second cycle based on clinical and radiological findings in accordance with the RECIST criteria.

### Pharmacodynamic and pharmacokinetic assays

These were performed in subset of patients. Samples for PBMC MGMT activity were obtained prior to treatment on day 1 of cycle 1, and at 2, 4, 6, 8 and 24 h after dosing in four patients. A total of 5–10 ml of venous blood was collected into tubes containing 100 *μ*l 0.5 M EDTA and stored on ice for a maximum of 4 h prior to isolation of PBMC and analysis of MGMT. For pharmacokinetics, two 5-ml venous blood samples were drawn predose, and at 0.5, 1, 2, 3, 4, 6 and 8 h after dosing on days 1 and 5 of cycle 1 in nine patients, for determination of lomeguatrib and TMZ concentrations according to previously published methods (Watson and Margison, 2000).

## RESULTS

Nineteen patients were recruited to the study between September 2003 and January 2004. All had stage IV colorectal cancer and had received prior chemotherapy, as described in Tables 1 and 2. Nine patients had one prior chemotherapy treatment for metastatic disease and nine had two previous regimens. A further patient had three prior chemotherapy treatments but was enrolled, as the first of these had been given 10 years previously, prior to resection of a hepatic metastasis. Five patients had undergone radiotherapy, as either adjuvant (three) or palliative (two) treatment. All but one patient had undergone surgery for their disease.

All the patients received treatment as defined in the protocol, although two individuals missed partial doses during cycle 1 and cycle 4 respectively. A further patient took all the medication due in cycle 1, but not according to the recommended schedule.

### Treatment efficacy

None of the patients responded to treatment. Three patients had stable disease: one each progressed after three and four cycles of treatment, and the other remained progression-free at the end of all six cycles of lomeguatrib and TMZ. No fall in CEA was observed in any patient. Median time to progression was 50 days (95% confidence interval 47–60 days).

### Safety

All of the patients experienced adverse events related to treatment (Table 3), but these were for the most part mild to moderate in severity with the exception of thrombocytopaenia and anaemia (Table 3). One patient experienced rectal bleeding while thrombocytopaenic and required a platelet transfusion. One other patient required a platelet transfusion, and growth factor support was administered to one patient due to ongoing grade IV neutropaenia.

One patient died while on treatment. He was admitted after receiving cycle 2 with increasing right upper quadrant pain, shortness of breath, dizziness and mild confusion. These were not considered related to study treatment and the patient was considered to be experiencing disease progression based upon a chest radiograph. He developed grade IV neutropaenia the following day, which was considered highly probable in relation to the study treatment, however, this was not treated due to the patient experiencing disease progression. He died 2 days later from disease progression and had no clinical evidence of infection at the time of death.

Forty-nine cycles of lomeguatrib and TMZ were delivered overall. Eleven cycles had to be delayed to allow recovery of neutropaenia and/or thrombocytopaenia, and two more to accommodate patients' domestic arrangements. Dose reductions in TMZ were required for nine patients (two reductions in one case), all as a consequence of haematological toxicity.

### Pharmacokinetics and pharmacodynamics

Pharmacokinetic samples for lomeguatrib (Table 4) and TMZ (data not shown) were obtained in nine randomly selected patients. No differences were apparent in parameters measured on day 1 as compared with those from day 5 for either drug. The data for TMZ were consistent with previous studies, including a recent phase II trial of the combination in melanoma (Ranson *et al*, 2006, 2007). *O*^6^-Methlylguanine-DNA alkyltransferase activity in PBMC in four patients fell rapidly after dosing with oral lomeguatrib with 92% depletion, compared with pretreatment values, at 2 h and no detectable activity at all subsequent time points was observed. *O*^6^-Methlylguanine-DNA alkyltransferase activity was not analysed in more patients as a similar study using lomeguatrib and TMZ in melanoma patients demonstrated the same results in over 40 patients (Ranson *et al*, 2007).

## DISCUSSION

The primary objective of the study was to evaluate the tumour response rate after administration of the combination of lomeguatrib and TMZ in patients with stage IV metastatic colorectal carcinoma. The original recruitment target was 30 patients, but the absence of responses coupled with evidence from other studies, which suggests that the dosing regimen of lomeguatrib was inadequate, led to the closure of the trial after 19 patients had been included (Ranson *et al*, 2006).

The best response was stable disease, but only one patient sustained this for the full 6 months of treatment. The majority of patients experienced disease progression at cycle 2, such that the median time to progression was only 50 days. Half of our patients were having their second line of therapy, where response rates of 20–30% are obtainable, and the other half their third or fourth line of treatment where responses are seen in 10–15%.

The adverse-effect profile of this combination of lomeguatrib and TMZ did not differ significantly from that associated with TMZ alone, although haematological toxicity was more pronounced. The majority of adverse events were mild (CTC grades I and II). All patients experienced adverse events of the gastrointestinal system, of which the majority were considered related to the study treatment. Blood dyscrasias were the second most frequently observed adverse event, and all were considered related to the study treatment. Of the grade III and IV adverse events, most were haematological. A general deterioration in performance status was observed in the patient population during the course of participation in the trial, consistent with patients with metastatic cancer whose disease was progressing.

The addition of lomeguatrib did not appear to alter this profile significantly, but it is likely to have triggered adverse haematological events at a lower dose of TMZ. This has been found in the original dose escalation phase I trial as well as in a comparative trial in melanoma (Ranson *et al*, 2006, 2007).

This study has demonstrated that the drug combination used was not efficacious in this indication but was safe with a comparable adverse event profile to TMZ alone with the exception of enhanced haematological toxicity. Potential reasons for this lack of efficacy include the wrong doses of TMZ and lomeguatrib and perhaps more importantly, the fact that colorectal cancer is not considered to be particularly sensitive to TMZ (Spiro *et al*, 2001). It may be that other downstream mechanisms of resistance are to blame. For example, deficient MMR leads to tolerance of methylation damage, and changes to the apoptotic machinery have been linked to TMZ resistance (Madhusudan and Middleton, 2005). In a melanoma study with the same regimen, tumour biopsy analysis suggested early recovery of MGMT activity, within 24 h, even when lomeguatrib doses were increased to 60 and 80 mg per day (Ranson *et al*, 2006). The methylation lesion at the *O*^*6*^ position in guanine leads to cell death only after one or two rounds of DNA replication. It therefore seems advisable in future to use higher doses of lomeguatrib than we have in this trial, and to continue dosing for some days following the last administration of TMZ.

Our findings are consistent with those from other trials involving MGMT pseudosubtrates (Friedman *et al*, 1998, 2000; Quinn *et al*, 2002; Gajewski *et al*, 2005; Ranson *et al*, 2006). In trials performed so far with *O*^6^-benzylguanine (*O*^6^-BG) and lomeguatrib, there has been enhancement of toxicity, particularly myelosuppression when used in combination with alkylating agents. Consequently, only about 20% of the standard dose of alkylating agent can be administered when combined with *O*^*6*^-BG, and with lomeguatrib, patients tolerate approximately two-thirds of the standard drug dose. To date, superior efficacy with this approach has not been demonstrated and it seems unlikely that increased responses to alkylating agents in most tumours will be seen while the problem of enhanced toxicity remains.

On the current evidence, further trials of lomeguatrib and TMZ in metastatic colorectal cancer are not warranted. However, MGMT depletion has been found to enhance the cytotoxicity of classic alkylators, such as cyclophosphamide and topoisomerase I inhibitors, such as irinotecan (Friedman *et al*, 1999, 2002). In the case of irinotecan, sensitivity to its active metabolite SN-38 is inversely correlated with MGMT expression in cell lines (Okamoto *et al*, 2002). Interestingly, there is schedule-dependent synergy between TMZ and irinotecan, likely mediated through the effects of *O*^*6*^-methylguanine on the kinetics of the interaction between topoisomerase 1 and DNA. *O*^6^-Methlylguanine-DNA alkyltransferase depletion further enhances the cytotoxicity of TMZ and irinotecan (Sabharwal and Middleton, 2006). A trial combining lomeguatrib and irinotecan in metastatic colorectal cancer is ongoing.

In conclusion, this combination of lomeguatrib and TMZ is not efficacious in metastatic colorectal cancer, which is probably a reflection of the insensitivity of colorectal cancer to TMZ. Results from an ongoing trial combining irinotecan and lomeguatrib will be interesting.

## Figures and Tables

**Table 1:** Patient characteristics

	***N*=19 (%)**
Median age, years (range)	59 (39, 81)
Male/female	14/5
	
*Site of primary tumour*	
Caecum	6 (32)
Colon	5 (26)
Rectum	4 (21)
Not specified	4 (21)
	
*Performance status (ECOG)*	
0	5 (26)
1	14 (74)
	
*Prior chemotherapy*	
Fluoropyrimidines	19 (100)
Irinotecan	15 (79)
Oxaliplatin	12 (63)
Others^a^	3 (16)

ECOG=Eastern Cooperative Oncology Group.

a Hydroxyurea/5-FU (1); low-dose cyclophosphamide/methotrexate/celecoxib (2).

**Table 2:** Details of previous chemotherapy treatments

Prior chemotherapies	*N*=19 (%)
5-fluorouracil	10 (52.6)
Capecitabine	1 (5.3)
Irinotecan	15 (78.9)
Folinic acid	12 (63.2)
Calcium folinate	3 (15.8)
Leucovorin	1 (5.3)
Leucovorin calcium	1 (5.3)
Oxaliplatin	12 (63.2)
Cyclophosphamide	2 (10.5)
Methotrexate	2 (10.5)
Celecoxib	1 (5.3)
Calcium levofolinate	1 (5.3)
Tyrosine hydroxylase inhibitors	1 (5.3)

**Table 3:** Adverse events considered to be related to study treatment

	**All adverse events *n*=19 (%)**	**G3/4 adverse events (%)**
Thrombocytopaenia	68.0	52.7
Neutropaenia	52.6	63.7
Febrile neutropaenia	5.3	5.3
Anaemia	21.1	10.5
Nausea	68.4	0
Vomiting	26.3	5.3
Constipation	52.6	21.1
Diarrhoea	21.1	0
Dyspepsia	5.3	0
Fatigue	26.3	0
Pyrexia	15.8	0
Anorexia	42.1	0
Headache	36.8	0
Alopecia	5.3	0

**Table 4:** Lomeguatrib pharmacokinetic data

	***T***_**1/2**_ **(h)**	***C***_**max**_ **(ng ml^−1^)**	***T***_**max**_ **(h)**	**AUC**_**(0–8)**_ **(ng ml^−1^ h)**	***V***_**ss**_ **/*F*^a^ (l)**	**CL/*F* (l h^−1^)**
*D1*						
*N*	7^b^	9	9	9	7^b^	7^b^
Mean	1.4	35.2	3.2	104.7	1555.7	386.7
s.d.	0.4	24.5	1.9	77.4	1235.1	238.6
Median	1.3	22.5	3.0	87.9	1301.8	342.1
Range	(1.0; 2.1)	(5.0; 71.9)	(2.0; 8.0)	(8.8; 223.9)	(661.5; 4157.6)	(171; 862.2)
						
*D5*						
*N*	9	9	9	9	9	9
Mean	1.6	40.5	3.1	114.6	1737.4	392.2
s.d.	0.7	24.4	1.1	56.9	1136.6	200.4
Median	1.4	36.9	3.0	118.7	1568.0	334.6
Range	(0.8; 3.2)	(12.2; 85.2)	(1.0; 4.0)	(43; 200.9)	(695.7; 4454.0)	(178.9; 739.8)

s.d.=standard deviation.

a *V*_ss_/*F*: Volume of distribution at steady state uncorrected for fraction absorbed.

b Samples were taken for nine patients; however, patient profiles did not allow all pharmacokinetic parameters to be calculated for all patients.
