# A phase II trial of lomeguatrib and temozolomide in metastatic colorectal cancer

**DOI:** 10.1038/sj.bjc.6605234

**Published:** 2009-07-28

**Authors:** O A Khan, M Ranson, M Michael, I Olver, N C Levitt, P Mortimer, A J Watson, G P Margison, R Midgley, M R Middleton

**Correction to:** *British Journal of Cancer* (2008) **98**, 1614–1618; doi:10.1038/sj.bjc.6604366

In the original submission and subsequent publication of the above paper in the *British Journal of Cancer* last year, the authors failed to acknowledge the support of the ‘NIHR Biomedical Research Centre, Oxford’ in carrying out their study.

They are now grateful for the chance to correct this omission.

